# Does COVID-19 infection have an impact on children’s psychological problems?

**DOI:** 10.1186/s43045-021-00155-z

**Published:** 2021-10-27

**Authors:** Gellan K. Ahmed, Khaled Elbeh, Hamdy M. Gomaa, Saeed Soliman

**Affiliations:** 1 Department of Neurology and Psychiatry, Faculty of Medicine, Assiut University, Assiut, Egypt; 2 Department of Child and Adolescent Psychiatry, Institute of Psychiatry, Psychology and Neuroscience, King’s College London, London, SE5 8AF UK; 3 Department of Family Medicine, Faculty of Medicine, Assiut University, Assiut, Egypt; 4 Department of Family Medicine, Faculty of Medicine, Cairo University, Cairo, Egypt

**Keywords:** COVID-19, Children, Psychiatric comorbidity

## Abstract

**Background:**

Coronavirus disease 2019 (COVID-19) has a significant impact on children, adolescents, and their families. So, the purpose of this study is to investigate the prevalence of children’s psychological problems during the COVID-19 pandemic and their association of COVID-19 infection in children and their risk factors. A cross-sectional study was conducted on 148 children aged 6–12 years old categorized into 2 groups based on COVID-19 infection history. Participants were assessed by the Socioeconomic Scale and the Checklist for Children’s Behavior (CBCL).

**Results:**

Children who had COVID-19 had a high percentage of problems regarding family, school, social, financial, and parent problems due to the COVID-19 pandemic. Regarding CBCL, children who had COVID-19 infection had a higher percentage of clinical rating than the other group regarding withdrawal (11.1% vs. 8.9%), anxious/depressed (33.3% vs. 25%), somatic (11.1% vs. 10.7%), internalizing (61.1% vs. 48.2%), externalizing (38.9% vs. 35.7%), and total problems (50% vs. 44.6%). Family history of psychiatric disorder and the presence of three or more offspring were at high risk for internalizing problems, while those with school problems during pandemic were more vulnerable for internalizing and total problems.

**Conclusion:**

Children with COVID-19 infection had a higher risk of developing psychological problems, such as withdrawal, anxiety/depression, somatic, internalizing, externalizing, and total problems.

## Background

Coronavirus disease 2019 (COVID-19) has a significant impact on people all over the world. Isolation, contact limitations, and financial closure would all have a profound effect on the psychological environment of the impacted countries. Children, adolescence, and their families are severely affected by the current circumstances [1].

Children have a less chance of acquiring the severe form of the illness and need less hospitalization and mechanical breathing than adults [2]. However, children and adolescents may be particularly vulnerable to the pandemic’s biopsychosocial stressors. The quarantine strategy was activated to prevent virus spread. It disturbed people’s daily life routines due to social isolation and difficulty in understanding the outbreak’s short- and long-term consequences [3, 4].

During the COVID-19 pandemic, many factors impacted children and adolescents, including the closure of kindergartens and schools, the restriction of social contacts, and outdoor leisure activities. Parents were encouraged to help their children with home education while also working from home. Other family members’ and social support systems’ assistance has declined. Aside from worries and fears about COVID-19, the economic situation has deteriorated, escalating unemployment rates in all countries affected. This has placed a great stress on children, adolescents, and their families, potentially leading to distress, mental health issues, and violence [1].

COVID-19 pandemic consequences could be disastrous. As a child or adolescent’s central nervous system (CNS) is in a susceptible developmental time, any stressful issues during crucial time can cause short- and long-term physiological [5, 6], cognitive, and behavioral damages [5, 7]. The amygdala, hippocampus, and prefrontal cortex are the specific brain areas that are related to stress. The response is mediated by a complex series of hormonal, immune, and neurotransmitter responses, which can alter glial cell response patterns and monoamine metabolism, resulting in cell apoptosis and psychiatric problems [5, 8].

People with COVID-19 infection may have psychiatric problems caused by biological factors other than psychosocial factors [9], such as inflammation which is linked to the pathogenesis of depression [10], schizophrenia [11], and bipolar disorder [12]. In addition to direct infection, blood circulation, neuronal involvement, hypoxic injury, immune injury, and angiotensin-converting enzyme 2 (ACE2) binding are the potential causes of CNS damage that trigger psychiatric problems in survivors [13].

So, the purpose of this study is to investigate the psychological problems of the COVID-19 pandemic in children aged 6 to 12 years old. Also, the impact of COVID-19 infection and the possible risk factors for these problems.

## Methods

### Participants and procedures

From February to May 2021, a cross-sectional study was conducted. A total of 148 children aged 6–12 years old were recruited from three Assiut government primary schools, Egypt. During this time, students were only allowed to attend school for 2 days a week, and most of their education was provided online. So, with the permission of the manager’s school, we distributed an online Google form through school websites to invite parents to participate in our study to assess their children. The parents who accepted our invitation were interviewed with their children at Assiut University’s Child Psychiatry Clinic, the Neurology and Psychiatric Hospital. Furthermore, the children were divided into two groups: the first group who had COVID-19 infection (*N* = 36) as documented with a PCR report, and the second group, who did not have COVID-19 infection (*n* = 112). Children with a history of mental disorders, a medical or neurological disease, or their intelligence quotient (IQ) below 70 were excluded from the study.

### Measures

At the start of the study, participants were given a full psychiatric and medical history by semi-structured interview prepared by the researchers. Parents were asked about the previous infection of COVID-19 in their children with PCR report and the duration of the diagnosis.

#### Sociodemographic data

Sociodemographic data included age, gender, birth order, number of children, delivery type and problems, delay of speech development, delay of motor development, and family history of psychiatric disorders.

#### The impact of COVID-19 pandemic on children life

Information gathering include the impact of COVID-19 on children regarding (Did any one of the family (first relative degree) have a diagnosis as COVID-19 infection? (yes/no), Did any one of the family (first relative degree) die from COVID-19 infection? (yes/no), School problems (academic) due to COVID-19 infection? (yes/no), Social problems (peers) due to COVID-19 infection? (yes/no), Problems in parent relationship due to COVID-19 infection? (yes/no), Increase family expenses due to COVID-19 infection? (yes/no), Decrease family income due to COVID-19 infection? (yes/no).

#### The socioeconomic scale

The socioeconomic scale [14] is a tool for determining social burdens and socioeconomic classes. It also includes four major variables: the father’s and mother’s educational levels, their respective occupations, the total family income, and the family’s lifestyle.

#### The checklist for children’s behavior (CBCL)

The CBCL [15] is a questionnaire with 113 items that parents fill out to help identify emotional and behavioral problems in children and adolescents aged 6 to 18. It is graded on a three-point Likert scale ranging from 0 (no response) to 2 (complete response) (occurs often). The items are rated based on the child’s behavior over the previous 6 months.

### Statistical analysis

SPSS was used to conduct the statistical analysis (version 26). As descriptive statistics, frequencies and percentages were reported. The categorical variables were evaluated using the Pearson chi-squared test. To detect the difference in mean values between the two groups, the independent *t* test was used to assess quantitative variables. Researchers used multivariate logistic regressions to identify possible risk factors for externalizing, internalizing, and total problems in children. Statistical significance was defined as a probability value (*p* value) of less than 0.05.

## Results

A total of 148 child were assessed and included in this study of them, the majority were males, first birth order, no postpartum problems, no delay in speech or motor development, no family history of psychiatric disorders, and belong to middle socioeconomic level (see Table 1). Regarding COVID-19 infection status, 36 had COVID-19 infection as documented with a PCR report, while 112 did not have COVID-19 infection.

**Table 1:** Sociodemographic data of studied groups

Variables	Children had COVID-19 (***N*** = 36)	Children did not have COVID-19 (***n*** = 112)	Total of participants (***N*** = 148)	***P*** value
**Age (years) (mean ± SD)**	9.11 ± 1.9	8.13 ± 1.7	8.36 ± 1.8	0.001*
**Gender**				
Males	20 (55.6%)	58 (51.8%)	78 (52.7%)	0.7
Females	16 (44.4%)	54 (48.2%)	70 (47.3%)	
**Order of birth**				
First	24 (66.7%)	82 (73.2%)	106 (71.6%)	0.66
Second	10 (27.7%)	22 (19.6%)	32 (21.6%)	
Third or more	2 (5.6%)	8 (7.1%)	10 (6.8%)	
**Number of children**				
Only child	2 (5.6%)	8 (7.1%)	10 (6.8%)	0.74
Two	14 (38.8%)	36 (32.1%)	50 (33.8%)	
Three or more	20 (55.6%)	68 (60.7%)	88 (59.4%)	
**Pregnancy problems**	12 (33.3%)	10 (8.9%)	22 (14.8%)	0.33
**Post-partum problems for children**	0 (0%)	4 (3.6%)	4 (2.7%)	0.32
**Delay of speech development**	16 (44.4%)	30 (26.8%)	46 (31.1%)	0.039*
**Delay of motor development**	2 (5.6%)	10 (8.9%)	12 (8.1%)	0.4
**Family history of psychiatry disorders**	4 (11.1%)	20 (17.9%)	24 (16.2%)	0.24
**Socioeconomic level**				
Low class	4 (11.1%)	18 (16.1%)	22 (14.9%)	0.03*
Middle class	24 (66.7%)	86 (76.8%)	110 (74.3%)	
High class	8 (22.2%)	8 (7.1%)	16 (10.8%)	

*Significant *p* value

### Sociodemographic data

Among the studied groups, there was a significant difference regarding age, speech development delay, and socioeconomic status. The mean age of children who had COVID-19 was 9.11 ± 1.9, while the mean age of children without COVID-19 infection was 8.13 ± 1.7. In the COVID-19 infection group, the mean time of diagnosis of COVID-19 infection was 2.13 ± 1.5 months before the interview.

Larger proportion of children who had COVID-19 (44.4%) had delay of speech development compared to children without COVID-19 infection (26.8%). Regarding the socioeconomic (SE) level, middle class was the prevailing in both groups. However, lager proportion of children who had COVID-19 was in the high SE class (22.2%) in comparison to children without COVID-19 infection (7.1%). Subsequently, children without COVID-19 infection had higher percentage of lower class (16.1%) when compared to children with history of COVID-19 infection (11.1%).

### The impact of COVID-19 pandemic on children life

In Table 2, children who had COVID-19 were more affected by the COVID-19 pandemic in their life than children without COVID-19 infection regarding family member who got an infection or died from COVID-19; school, social, parent problems; increase family expenses; and decrease family income.

**Table 2:** Association between COVID-19 infection status and areas of children’s life

Variables	Children had COVID-19 (***N*** = 36)	Children did not have COVID-19 (***n*** = 112)	Total of participants (***N*** = 148)	***P*** value
Did any one of the family (first relative degree) have diagnosis as COVID-19 infection.	36 (100%)	38 (33.9%)	74 (50%)	0.000*
Did any one of the family (first relative degree) die from COVID-19 infection.	4 (11.1%)	6 (5.4%)	10 (6.8%)	0.2
School problems (academic) due to covid19	24 (66.7%)	72 (64.3%)	96 (64.9%)	0.48
Social problems (peers) due to COVID-19	31 (86.1%)	96 (85.7%)	127 (85.8%)	0.05
Problems in parent relationship due to COVID-19	28 (77.7%)	86 (76.8%)	114 (77%)	0.27
Increase family expenses due to COVID-19	14 (38.9%)	40 (35.7%)	54 (36.5%)	0.43
Decrease family income due to COVID-19	14 (38.9%)	38 (33.9%)	52 (35.1%)	0.36

*Significant *p* value

### CBCL result

According to the CBCL scores, the percentage of children who had a clinical rating of the profile of syndromes during the COVID-19 pandemic were 9.5% of withdrawal; 13.5% of somatic complaints; 14.9% of social problems; 27% of anxious/depressed; 18.9% of thought problems; 54.1% of rules breaking behavior; 18.9% of aggressive behavior; 23% of depression problems; 24.3% of anxiety problems; 10.8% of somatic problems; 18.9% of oppositional defiant problems; 17.6% of conduct problems; 51.4% of internalizing problems; 36.5% of externalizing problems; and 45.9% of total problems.

There was a significant difference regarding thought problems and rules breaking behavior between the studied groups. Children who had COVID-19 had a higher percentage of clinical rating than children without COVID-19 infection regarding withdrawal (11.1% vs. 8.9%), anxious/depressed (33.3% vs. 25%), internalizing problems (61.1% vs. 48.2%), externalizing problems (38.9% vs. 35.7%), and total problems (50% vs. 44.6%).

In addition, the CBCL Profile of DSM-5 scales had a higher percentage of clinical rating in children who had COVID-19 than without COVID-19 infection regarding depression problems (27.8% vs. 21.4%) and somatic problems (11.1% vs. 10.7%) (see Tables 3 and 4).

**Table 3:** CBCL Profile of syndromes of studied groups

Variables	Children had COVID-19 (***N*** = 36)	Children did not have COVID-19 (***n*** = 112)	Total of participants (***N*** = 148)	***P*** value
**Withdrawn**				
Normal	*26* (*72.2%*)	92 (82.2%)	*118* (*79.7%*)	*0.37*
Borderline	6 (16.7%)	10 (8.9%)	16 (10.8%)	
**Clinical rating**	4 (11.1%)	10 (8.9%)	14 (9.5%)	
**Somatic complaints**				
Normal	28 (77.8%)	80 (71.4%)	108 (73%)	0.75
Borderline	4 (11.1%)	16 (14.3%)	20 (13.5%)	
**Clinical rating**	4 (11.1%)	16 (14.3%)	20 (13.5%)	
**Social problems**				
Normal	30 (83.3%)	90 (80.3%)	120 (81%)	0.68
Borderline	2 (5.6%)	4 (3.6%)	6 (4.1%)	
**Clinical rating**	4 (11.1%)	18 (16.1%)	22 (14.9%)	
**Anxious/depressed**				
Normal	*16* (*44.4%*)	62 (55.4%)	*78* (*52.7%*)	*0.49*
Borderline	8 (22.2%)	22 (19.6%)	30 (20.3%)	
**Clinical rating**	*12* (*33.3%*)	28 (25%)	40 (27%)	
**Thought problems**				
Normal	*30* (*83.3%*)	72 (64.3%)	*102* (*68.9%*)	0.003*
Borderline	*0* (*0%*)	18 (16.1%)	*18* (*12.2%*)	
**Clinical rating**	*6* (*16.7%*)	22 (19.6%)	*28* (*18.9%*)	
**Attention problems**				
Normal	*36* (*100%*)	104 (92.9%)	*140* (*94.6%*)	*0.12*
Borderline	*0* (*0%*)	8 (7.1%)	*8* (*5.4%*)	
**Clinical rating**	*0* (*0%*)	*0* (*0%*)	*0* (*0%*)	
**Rules breaking behavior**				
Normal	*14* (*38.9%*)	44 (39.3%)	*58* (*39.2%*)	0.03*
Borderline	6 (16.7%)	4 (3.6%)	10 (6.8%)	
**Clinical rating**	16 (44.4%)	64 (57.1%)	80 (54%)	
**Aggressive behavior**				
Normal	24 (66.7%)	72 (64.3%)	96 (64.9%)	0.25
Borderline	8 (22.2%)	16 (14.3%)	24 (16.2%)	
**Clinical rating**	4 (11.1%)	24 (21.4%)	28 (18.9%)	
**General profile of problems**				
**Internalizing problems**				
Normal	8 (22.2%)	42 (37.5%)	50 (33.8%)	0.23
Borderline	6 (16.7%)	16 (14.3%)	22 (14.9%)	
**Clinical rating**	22 (61.1%)	54 (48.2%)	76 (51.3%)	
**Externalizing problems**				
Normal	14 (38.9%)	54 (48.2%)	68 (45.9%)	0.55
Borderline	8 (22.2%)	18 (16.1%)	26 (17.6%)	
**Clinical rating**	14 (38.9%)	40 (35.7%)	54 (36.5%)	
**Total problems**				
Normal	12 (33.3%)	44 (39.3%)	56 (37.8%)	0.8
Borderline	6 (16.7%)	18 (16.1%)	24 (16.2%)	
**Clinical rating**	18 (50%)	50 (44.6%)	68 (45.9%)	

*Significant *p* value

**Table 4:** CBCL Profile of DSM-5 scales of studied groups

Variables	Children had COVID-19 (***N*** = 36)	Children did not have COVID-19 (***n*** = 112)	Total of participants (***N*** = 148)	***P*** value
**Depression problems**				
**Normal**	16 (44.4%)	66 (59%)	82 (55.4%)	*0.31*
**Borderline**	10 (27.8%)	22 (19.6%)	32 (21.6%)	
**Clinical rating**	10 (27.8%)	24 (21.4%)	34 (23%)	
**Anxiety problems**				
**Normal**	18 (50%)	68 (60.7%)	86 (58.1%)	0.02*
**Borderline**	12 (33.3%)	14 (12.5%)	26 (17.6%)	
**Clinical rating**	6 (16.7%)	30 (26.8%)	36 (24.3%)	
**Somatic problems**				
**Normal**	28 (77.8%)	84 (75%)	112 (75.7%)	*0.88*
**Borderline**	4 (11.1%)	16 (14.3%)	20 (13.5%)	
**Clinical rating**	4 (11.1%)	12 (10.7%)	16 (10.8%)	
**Attention deficit/hyperactivity problems**				
**Normal**	36 (100%)	104 (92.9%)	140 (94.6%)	0.1
**Borderline**	0 (0%)	8 (7.1%)	8 (5.4%)	
**Clinical rating**	0 (0%)	0 (0%)	0 (0%)	
**Oppositional defiant problems**				
**Normal**	34 (94.4%)	82 (73.2%)	116 (78.4%)	0.009*
**Borderline**	0 (0%)	4 (3.6%)	4 (2.7%)	
**Clinical rating**	2 (5.6%)	26 (23.2%)	28 (18.9%)	
**Conduct problems**				
**Normal**	18 (50%)	72 (64.2%)	90 (60.8%)	0.13
**Borderline**	12 (33.3%)	20 (17.9%)	32 (21.6%)	
**Clinical rating**	6 (16.7%)	20 (17.9%)	26 (17.6%)	

*Significant *p* value

### Identification of the possible risk factors of psychiatric comorbidity of COVID-19 pandemic in children

In Table 5, the multivariate logistic regression module regarding risk factors for externalizing, internalizing, and total problems of the studied groups. Surprisingly, none of the studied risk factors were associated with externalizing problems. Children with a family history of psychiatric disorder, and the presence of three or more offspring were more vulnerable to internalizing problems while children who had school problems during pandemic were more vulnerable to internalizing and total problems.

**Table 5:** Multivariate logistic regressions analysis of risk factors for internalizing, externalizing, and total problems in children

Variable	Internalizing problems		Externalizing problems		Total problems	
	***P*** value	Odds ratio	***P*** value	Odds ratio	***P*** value	Odds ratio
**Gender**						
Females^a^	0.159	2.867	0.233	0.561	0.426	0.732
**Having COVID-19 infection**	0.860	1.158	0.798	1.189	0.802	0.836
**Number of children**^**b**^						
Two	0.868	0.777	0.283	3.141	0.114	6.523
Three or more	0.000*	15.858	0.280	1.731	0.148	2.212
**Order of birth**^**c**^						
Second	0.051	0.093	0.232	2.929	0.144	0.201
Third or more	0.099	0.119	0.733	0.720	0.131	0.172
**Delay of speech development**	0.066	0.305	0.139	0.440	0.186	0.465
**Delay of motor development**	0.898	1.154	0.175	4.043	0.064	9.274
**Family history of psychiatric disorders**	0.013*	1.051	0.936	0.947	0.989	1.010
**Total score of socioeconomic level**	0.090	0.469	0.182	0.593	0.127	0.522
Have family infection	0.053	0.222	0.160	0.441	0.670	1.300
Have died family member	0.496	2.040	0.747	0.715	0.354	0.368
School problems	0.004*	1.122	0.067	1.366	0.033*	1.274
Social problems	0.346	2.274	0.878	0.904	0.446	1.795
Problems in parent relationship	0.105	4.532	0.829	1.128	0.609	1.368
Increase family expenses	0.413	0.460	0.068	0.260	0.074	0.190
Decrease family income	0.178	0.305	0.167	2.552	0.467	1.775

^a^Male gender was the reference

^b^The only child was the reference

^c^The first child was reference

*Significant *p* value

## Discussion

The important findings in this study are a higher percentage of clinical rating in children who had COVID-19 compared to children who did not have COVID-19 regarding internalizing problems (61.1% vs. 48.2%), externalizing problems (38.9% vs. 35.7%), and total problems (50% vs. 44.6%). No identified risk factors were associated with externalizing problems. At the same time, children who had a family history of psychiatric disorder, and the presence of three or more offspring were more vulnerable to internalizing problems while children who had school problems during pandemic were more vulnerable for internalizing and total problems.

In the current study, children who had COVID-19 were more frequent in speech development delay, belonged to a high socioeconomic level and more suffered regarding family members who got an infection or died from COVID-19; school, social, and parent problems; increase family expenses; and decrease family income than the other group.

Social contact plays a major role in development of cognition, emotions, attachment, and relationships [16]. Also, it helps in the physiological regulation of the body’s responses to acute stressors [17]. Furthermore, Danese et al. (2009) found that childhood social isolation is a risk factor for depression in adulthood [5]. Since dysfunctional systemic inflammation has a role in impaired neurodevelopmental processes and causes cognitive and mood disturbances [18], it seems reasonable to assume that inflammatory changes in children exposed to COVID-19 infection could causes long-term physiological and psychological harm and should be a major concern [19].

Fear’s influence on children and adolescents is another major source of concern. During COVID-19 pandemic, a study about fear in the quarantined children and adolescents found a high prevalence of fear among the studied groups. This fear was primarily related to financial concerns, risk of COVID-19 infection, and infecting others [20]. Furthermore, it is unsurprising that adolescence is a stress-sensitive developmental period in terms of fear regulation, chronic stress has been shown to have a significant impact on amygdala prefrontal cortex connectivity and activity, impairing, for example, fear memory extinction [21]. So, more research would be needed to study the negative effects of fear on the developing brain during COVID-19 pandemic [19].

Also, school routines are important coping mechanisms for children and adolescent with psychiatric problems [22]. Moreover, absences from school have been linked to decreased physical activity, increased internet surfing, impaired sleep rhythm, and less nutritionally appropriate diets in children and adolescents [23].

Regarding socioeconomic level, low socioeconomic status (SES) has been consistently associated with adverse health outcomes and an increased risk of death [24–26]. According to cross-sectional studies, evidence suggests that inflammation and immunosenescence play a role as mediators in the relationship between socioeconomic status and health [27, 28]. Psychosocial stress such as adversity, trauma, or discrimination tends to have less self-mastery and social support [26, 29]. These stressors are thought to be a mediating factor in the poor health–low SES link. It may do so (at least in part) via weakened immune system [30, 31]. During the COVID-19 epidemic, lockdown curfews, self-isolation, social distance, and quarantine impacted total physical, mental, spiritual, and social well-being [32]. The current study reviewed a sample of children with COVID-19 to see if socioeconomic status (SES) was linked to immune function. Surprisingly, high socioeconomic children were more vulnerable to have COVID-19 infection.

In this study, according to CBCL, half of children with COVID-19 had psychiatric problems and had a higher percentage of clinical rating regarding withdrawal, anxious/depressed, somatic problems, internalizing problems, externalizing problems, and total problems than children who did not have COVID-19.

Two previous researches were done in China to evaluate psychiatric problems in children and adolescent, the first one was conducted on the quarantined children and adolescent and found them to have depressions, anxiety, or both while the second study was conducted during the pandemic and found symptoms of inattention, clinging, worry, and irritability [33, 34]. Another study was done in India to evaluate the quarantined children and adolescents, and found a high prevalence of psychological distress associated with experienced feelings of helplessness, worry, and fear [20].

A literature review reported the impacts of COVID-19 pandemic stressors on children and adolescents’ life. The COVID-19 pandemic stressor caused dysregulation in hypothalamic-pituitary-adrenal (HPA) axis by having inflammatory mediators’ synthesis and release, e.g., proinflammatory cytokines, neurotransmitters, hormones, and other molecules that can interfere with different physiological mechanisms during the early stages of life when the child or adolescent is exposed to situations of long-term stress. This imbalance could be linked to immune, endocrine, and nervous system overload and the risk of developing psychiatric disorders later in life. The possible short-term effects of exposure to COVID-19 pandemic stressors at this stage of life were distress and hopelessness, irregular food intake, abuse and trauma, interpersonal and environmental restraint, sensory deprivation, and neglect. In contrast, the possible long-term consequences were underdeveloped brain circuitry, obesity, etc. [19].

This study has a few important implications for policymakers, which may be applied to enhance families’ support with COVID-19 infection, as the infected children suffer more and are at an increased risk of developing psychiatric problems. As a result, psychiatric screening for children should be recommended, especially after infection with COVID-19. Psychoeducational programs are required to raise school, family, and community awareness of those children.

The current study had some limitations, including small sample size. As a result, we recommend that future studies with large representative samples to be conducted to confirm our findings. In addition, because most of the children were treated at home, we were unable to investigate the link between the severity of COVID-19 infection according to WHO guidelines [35] and psychological problems in children. Finally, due to the survey’s cross-sectional design, it was difficult to investigate causal relationships between variables. As a result, a longitudinal study should be considered.

## Conclusion

Children infected with COVID-19 had a higher risk of developing psychological problems, such as withdrawal, anxiety/depression, somatic problems, internalizing problems, externalizing problems, and total problems. Furthermore, they were more affected by the COVID-19 pandemic in terms of family member infection or death from COVID-19; school, social, and parent problems; increased family expenses; and decreased family income than children who did not have with COVID-19. Also, children with a family history of psychiatric disorder, school problems, and the presence of three or more offspring were more vulnerable to internalizing problems while children who had school problems were more vulnerable to total problems.

## Data Availability

All data generated or analyzed during this study are available from the corresponding author on reasonable request.

## Abbreviations

COVID-19: Coronavirus disease 2019
CNS: Central nervous system
ACE2: Angiotensin-converting enzyme 2
IQ: Intelligence quotient
CBCL: The Checklist for Children’s Behavior
SES: Socioeconomic status
